# To Be or Not to Be

**Published:** 1887-05

**Authors:** James G. Palmer


### "TO BE OR NOT TO BE."

BY DR. JAMES G. PALMER.

The Central Dental Association of Northern New Jersey had a "grand talk" at its regular meeting, March 21st. The invitations gave a list of those high in our profession who were to speak on Dr. Kingsley's paper, which was the first thing on the programme, and was entitled "The Present Relation of Dentistry to Medicine." Most of those invited were on hand, but strange to say the discussion was altogether one-sided.

Dr. Kingsley's paper was based upon the one read before the New England Dental Society, which has provoked so much discussion. In substance he said: "Medicine in its broad signification is applied to the entire healing art, and comprehends everything under the sun for the relief as well as the prevention of physical suffering. In the popular mind, however, 'medicine,' and 'medical practice' mean the practice of a physician, and as such they are only one branch of the healing art. No one disputes that dentistry is also a branch of the same healing art. Dentistry and dental surgery are constantly used as synonymous terms. Dentistry is a great deal more than dental surgery. The latter is simply one branch of dentistry, and is that which, if taught in medical schools, would properly become a specialty of surgery.

"I have heard it gravely stated before a dental society that 'there are but three professions, law, theology and medicine.' It is absurd in this day of progress to say that no new profession can arise, and that every new development of science must of necessity come within the scope and become a specialty of one or other of these three professions. I want to reaffirm the declaration that 'dentistry in America is practically an independent profession, and not subordinate to any other,' and to maintain that declaration I now devote myself."

After referring to the many dental colleges, their graduates, the studies taught, their certificates, etc., he said, "If dentistry had any distinct parentage, it was not in medicine. We recall the historic fact that it originated with barbers, and in no sense with the medical profession. Because medical graduates have adopted dentistry as a vocation, does not make it a specialty in medicine. One of the most distinguished of living dentists stepped out of a gun-smith's shop into dentistry. Another prominent one was once a tailor, and still another served his apprenticeship in a wagon shop, and not one of these had an intermediate medical training. What reciprocal interest is there between the practitioners of medicine and the practitioners of dentistry?

With reference to the recent anniversary meeting of the First District Society, he said that of the five thousand M. D.'s in New York City and vicinity, not one, so far as he could learn, was present, nor has he been able to find the slightest allusion to it in any medical journal. After some eulogium of the medical profession, he referred to the coming Medical Congress in this wise: "The section is no longer proscriptive. If it is the policy of the management to admit all reputable dentists, irrespective of special invitations, the whole profession will rejoice." He closed by saying, in regard to the proposed International Dental Congress, that "it is but justice to those interested in this movement that certain reports be corrected. It has been stated that this movement was hostile to the dental section of the Medical Congress. Nothing could be farther from the truth. There is no hostility on the part of the American Dental Association toward that section in the Medical Congress, nor is there ever likely to be."

Following Dr. Kingsley came Dr. J. Foster Flagg with a capital address. He asked to defer his paper till later, and Dr. C. S. Stockton was called upon as the representative of the opposition.

His first statement, however, showed decidedly that he was "on the fence." "I do not know," said he, "that I am on the other side; you take that for granted. I have never taken any special position one way or the other, in regard to this subject." He spoke of Dr. Kingsley's paper as appealing to the vain glory that is in ourselves, and as it had appealed to our baser interests it was scarcely worth his while to say much. "Will all that has been said, or that may be said, change the status of dentistry to-day?

No. We will go on just as we have gone on. The men who started out humbly in dentistry, if they did wrongly did no especial harm to anybody. Those who were of very meagre knowledge, who knew scarcely anything about dentistry or anything else, entered into it and in a measure established dentistry, and then in a little while came into it men who were not ignorant, and they established dentistry upon a better plane and made it what it is. The more a man knows, the better dentist he will be. I claim that a man who has been educated to administer remedies, no matter of what kind, for the relief or cure of the diseases of human beings, and who practices on the strength of that education, is certainly practicing medicine as much as it is possible for any human being to do so."

Following Dr. Stockton came Dr. W. H. Dwinelle. As a veritable M. D., one entitled to it by reason of study and passing an examination, as well as by reason of his years, his position commands respect. Briefly, he referred to "the hope and expectation of many, if not a very large proportion of the founders of our profession, of Harris, of Townsend, of Hayden, of Hudson, of the Parmlys and others, that the day would come, which I believe is now dawning, when our profession would be regarded as an independent one, and not a specialty of medicine. Dentistry is composite, broad, and far-embracing in its character. With many other departments of art and science and mechanics, it includes medicine, but it is not included in or absorbed by medicine, nor is it a specialty of it, any more than it is a specialty of the many supplementaries which contribute to its establishment. It is no argument against us to say that as we could not do without medicine, therefore we are specialists in it. We may apply the same argument to chemistry, histology, mechanics, metallurgy, etc."

He gave way to Dr. Flagg, who was welcomed uproariously. (The ladies graciously smiled their approval, for they were well represented.)

He first went back some fifty years, for he said that Dr. Dwinelle's remarks had carried him to reminiscences of Harris, Hayden, Westcott, and the Parmlys, "when those gentlemen expressed the idea that, as in fact we were nothing, it behooved us to become something; and I think we have. I don't know that I can say anything about the present relation of dentistry to medicine, because I cannot see any. This thing was once worked over by the American Medical Association, and they said there was nothing in it.

Then followed his paper on "Dentistry *more* than a specialty in medicine." He spoke of what has already been alluded to, the decision of the M. D.'s that "dentistry did not pertain to medicine," —of the rapidity and resistlessness of its advance to its position among the distinctive callings of the day, of its being unlike in any particular any other profession, that all attempts to incorporate the teaching of dentistry with that of medicine has proved utterly abortive, and of the fact that every university or medical college which had attempted the work had found it "expedient" (their own word) to confer as the mark of a so-called "specialty"—the distinctive title of D. D. S. or D. M. D. He paid his respects to the dental section of the Medical Congress in an emphatic manner, and returning to the main question said in substance, that dentistry is said to be a branch of medicine. When claims of branchhood were officially investigated by the so-called parent-tree, the verdict was against them. This was accepted, and dentistry threw out roots, gained a trunk, sent out branches, and grew to become a tree of fair proportions. This attribute of root development is strangely overlooked, and all the famous growth that comes from it is quietly ignored. Medicine and dentistry are akin, because both work for suffering humanity. But does that make of dentistry a "specialty."

Anatomy! Of what avail are pelvic measurements? Why should a dentist be drilled exhaustively in studying the muscles of the legs and arms? But such anatomy as dentists need they should know thoroughly; the more, thus far, the better dentists.

Physiology! A fair amount of general physiology is quite sufficient, but *dental* physiology they cannot know too well. Do they get dental physiology from medicine? Far from it. And so with chemistry and metallurgy.

Materia medica! There we can but laugh; the United States Dispensatory enumerates eight or ten thousand salts, roots, tinctures, ointments, etc., etc., to dentistry practically worthless. Not a tincture is prepared as dentistry would prepare it. Pastes, tinctures, solutions and compounds in daily use by dentists are not mentioned, and yet on knowledge of all these depend the comfort of the patients and the success of the practitioners of dentistry.

There is in every dental class that graduates, men who will teach dentistry until all thoughts of any dependence, except independence, shall have been forgotten.

This is but a brief outline of a most excellent and well written paper, view it from whatever standpoint one will.

Dr. John B. Rich then followed, beginning also with reminiscences of fifty years ago. Among the many good things he said we remember the following: "When dentistry has established itself as a distinct science, who are they that claim that medicine is the parent of dentistry? Not the men who practice medicine, but a few dentists who are ashamed of the profession by which they earn their living.

Dr. W. H. Atkinson was then called on, and after he had gone over the subject, in his own "Atkinsonian" way, said he was "with and against every speech that had been made."

Dr. E. S. Niles, of Boston, was called for. He was the only one, if my memory serves me right, who recognized the presence of the ladies of our profession and said, "ladies and gentlemen." He had not considered the subject so much since the New England meeting as he had this evening, and must take the medical side of the question. "We agree that dentistry is a healing art." Taking the studies of both professions, he drew the conclusion that dentistry must be an outgrowth of the medical profession; among other things he said, "who first dissected the fifth pair of nerves? Was it a member of the dental profession? Upon what is based our knowledge of pathology? Is it upon works in dental literature or in medical literature? I have said the medical profession have need of us; they are ignorant of a great many points on which we could enlighten them. They have made a great many cruel thrusts at us indirectly; but the time is coming when they cannot afford to do it; yea, it has now come."

Thus ended a memorable meeting for Jersey, and apparently the end is not yet.

